# Edible Mushrooms and Beta-Glucans: Impact on Human Health

**DOI:** 10.3390/nu13072195

**Published:** 2021-06-25

**Authors:** Chiara Cerletti, Simona Esposito, Licia Iacoviello

**Affiliations:** 1 Department of Epidemiology and Prevention, IRCCS NEUROMED, 86077 Pozzilli, Italy; simona.esposito@moli-sani.org (S.E.); licia.iacoviello@moli-sani.org (L.I.); 2 Department of Medicine and Surgery, Research Center in Epidemiology and Preventive Medicine (EPIMED), University of Insubria, 21100 Varese-Como, Italy

**Keywords:** mushrooms, β-glucans, immune modulation, cancer adjuvant, microbiota, cardiometabolic system, healthy diet

## Abstract

Mushroom cell walls are rich in β-glucans, long or short-chain polymers of glucose subunits with β-1,3 and β-1,6 linkages, that are responsible for the linear and branching structures, respectively. β-glucans from cereals, at variance, have no 1,6 linkages nor branching structures. Both immunomodulatory and anti-inflammatory effects of mushrooms have been described using purified β-glucans or fungi extracts on cellular and experimental models; their potential clinical use has been tested in different conditions, such as recurrent infections of the respiratory tract or complications of major surgery. Another promising application of β-glucans is on cancer, as adjuvant of conventional chemotherapy. β-glucans may protect the cardiovascular system, ameliorating glucose, lipid metabolism, and blood pressure: these activities, observed for oat and barley β-glucans, require confirmation in human studies with mushroom β-glucans. On the other hand, mushrooms may also protect the cardiovascular system via a number of other components, such as bioactive phenolic compounds, vitamins, and mineral elements. The growing knowledge on the mechanism(s) and health benefits of mushrooms is encouraging the development of a potential clinical use of β-glucans, and also to further document their role in preserving health and prevent disease in the context of healthy lifestyles.

## 1. Introduction

Edible mushrooms are tasty and nutritious macro-fungi with fruiting bodies and mycelia, both spontaneous or cultured, belonging to the classes of Basidiomycetes and Ascomycetes. They represent a very small part of the about 150,000–160,000 species estimated to be present on earth. Within the 2000 species known as safe, although not all edible, 700 have reportedly pharmacological properties. Besides the pre-historical finding in the Italian Alps of a mushroom in possession of the Ice Man mummy discovered in 1991, possibly as a laxative for intestinal disease [1], the use of mushrooms as medicaments had already significantly spread in the cultures of ancient Greece, Rome, China, and India, and for centuries they have been part of the traditional oriental medicine: the health properties of these vegetables varied from dietary to nutraceutical, medical, and psychotropic effects [2].

Only in the last decades, however, scientific studies were dedicated to the comprehension of the biological mechanisms underlying mushroom beneficial effects. A recent PubMed search by “mushroom and health” as keywords, resulted in 2411 publications (on 21 April 2021); of these, almost 80 were reviews from 2020 to date. This underlines the actual great interest of nutritionists, biologists, and medical doctors on the scientific bases of mushrooms’ healthy properties.

In the present review we will focus on β-glucans as important constituents of mushrooms. Their beneficial health effects and related mechanisms are the main object of this review. β-glucans from yeast and cereals will be only considered as reference.

## 2. Chemical Structure of β-Glucans

Glucans constitute the cell wall of fungi and yeast, and are the main polysaccharides present in mushrooms, in addition to water (90%), proteins, and amino acids (10–40%), fats (2–8%), vitamins and antioxidants, residual salts, and metals. Total carbohydrates—including polysaccharides—are about 50% of the dry matter of mushrooms. 

**Figure 1:**
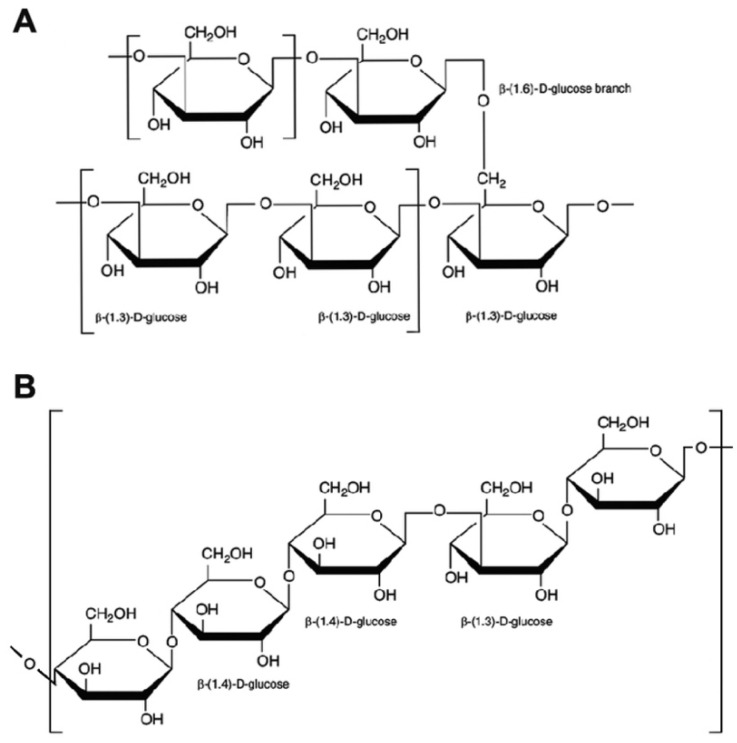
Chemical structures of β-glucans. The side branches of mushroom β-glucans (**A**) are attached to the β-1,3-D-glucan main chain by a β-1,6 linkage, and may consist of one or several β-D-glucose units; cereal β-glucans (**B**) are linear and consist of D-glucose molecules linked by 1,3 β- and 1,4 β- linkages.

The macromolecular structure of mushroom β-glucans consists of D-glucose monomers linked by β type glycosidic linkages at two differing positions, 1,3 and 1,6, responsible for the linear structure and of the main branching, respectively; in the α-glucans, proportionally less frequent in mushrooms, the polymers contain α type links. β-glucans from yeast have similar structures to those from fungi, the 1,3 linkages being more frequent (about 85%), in respect to the 1,6. Cereals are another important food source of β-glucans, which have 1,3 and 1,4 but no 1,6 linkages and branching structures. Though all β-glucans are similar in terms of type of glycosidic bonds linking together the glucose molecules, the structural build-ups of β-glucans varies in chain lengths, glycosidic bond positions, degree of branching, and three-dimensional structures (Figure 1).

Structural and physical characteristics of β-glucans vary from different species, cultivars, and also from growing environments, drying conditions of fungi and isolation/extraction methods of the β-glucans, with consequent important changes in the intensity and type of activities.

## 3. Pharmacokinetics of β-Glucans

A recently published study [3] reported the pharmacokinetics of lentinan, the β-glucan isolated from the fruiting body of shiitake mushroom (*Lentinula edodes*) after intravenous administration of 32 mg/kg of fluorescent labelled lentinan to rats. The circulating plasma levels of lentinan decreased with an apparent biphasic elimination; tissue distribution showed that most of the administered lentinan was concentrated in the liver, followed by kidney, spleen, heart, stomach, and intestine. The slow degradation of lentinan in the liver, recovered up to seven days, was confirmed by liver perfusion methods and liver microsome assays, which indicated that CYP450 enzymes (mainly CYP2D6 and CYP2C9) and epoxide hydrolase are involved in the metabolic degradation of this polysaccharide in the liver. These data confirm and extend previous pharmacokinetic studies in rats and other animal species by the use of radio labelled lentinan [4]. Pharmacokinetic studies of lentinan (as of other β-glucans) are limited by the lack of a specific detection method: lentinan was usually detected by the Limulus amebocyte lysate test (G-test) based on its typical triple helix conformation [5], but possibly disturbed by endotoxins or by other β-glucans. β-glucans, probably of dietary origin, are detectable in serum of healthy subjects with levels below 60 pg/mL; raised levels are a marker of invasive fungal infections, detectable by commercially available assays [6,7].

The pharmacokinetics of lentinan was detected by the Limulus test in the blood of 10 healthy volunteers and of three patients with advanced gastric cancer: peak plasma levels (50–70 ng/mL) were reached at the end of a 2h intravenous infusion of 1–4 mg of lentinans, and declined slowly over seven days, mainly due to uptake or degradation in the liver [8].

No reports on the absorption and pharmacokinetics of orally administered β-glucans are available, even though there are several reports of their biological effects. As specified below in the paragraph “β-glucans in metabolic and gastro-intestinal system”, mushroom β-glucans are not digested in the human gastro-intestinal tract, reaching the bowel virtually unchanged; β-glucans form a gel at the mucosa surface, which modulate biliary salt resorption, and modify the intestinal microbiota. In addition, small and large fragments of β-glucans found in the serum indicate that they may also be absorbed from the intestinal tract [9]. Macrophages within the Payer’s patches in the intestine physically transport the insoluble whole glucan particles into the gut-associated lymphoid tissue.

## 4. β-Glucans Biological Effects

Within the β-glucan health-related effects, the most studied and characterized are the modulation of the immune system and metabolic and gastrointestinal effects. Han et al. [10] carefully reviewed the information on structure–function relationship of β-glucans in relation to cellular immune modulation and antitumor activities: molecular weight, degree of branching, length of the sidechains, conformation of sugar residues, and consequent tridimensional structure, degree of solubility, and particulate size appear to be the most important features of β-glucans immune recognition, although some literature data are often inconsistent or contradictory. 

### 4.1. Immunomodulatory and Anti-Inflammatory Effects

β-glucans ingested as food or supplement reach the small intestine without any gastric digestion; they are then internalized by the intestinal epithelium and/or by the macrophages and presented to the immune cells within the Peyer’s patches in the intestine, and in distal lymphoid organs. Specific receptors of β-glucans are the Toll-like (TLR) and the C-type lectin-like receptors: within the latter, dectin-1 is the best characterized one and is predominantly expressed on the surface of monocyte/macrophages, neutrophils, and dendritic and natural killer (NK) cells [11,12]. An intracellular signal transduction follows the receptor recognition step and results in activation of the nuclear factor k-light-chain-enhancer of activated B cells (NF-kB), transcription of inflammatory-immune genes, production of cytokines, nitric oxide (NO), and reactive oxygen species (ROS) [2,13,14]. Other pattern recognition receptors (PRR) are implicated in β-glucan recognition and may act in cooperation with dectin-1/TLR [15], or even directly [16]. During injury, β-glucan receptors may be blocked, but a strong immunomodulatory response, independent of these receptors, can be stimulated, for instance, by β-glucans from a fungal pathogen, with expression of high levels of the interleukin-1 receptor antagonist (IL-1RA) [16]. Lentinan has been shown to upregulate cytokines in mouse macrophages and to attenuate IL-1β secretion resulting from Listeria activation of the absent in melanoma 2 (AIM2) inflammasome. Importantly, lentinan was able to reduce endotoxin lethality in mice despite the up-regulation of cytokine expression, most probably via inhibition of non-canonical inflammasome activation [17]. Similarly, β-glucans from *Saccharomyces cerevisiae* were reported to protect against endotoxin-induced shock and organ injury in rats, although the precise mechanism was not established [18]. In this context, it is worth mentioning that in mice treated with indomethacin, a bacterial β-glucan extract (Sinofilan) may contribute to septic shock, which occurs through translocation of enterobacterial flora to various organs, and systemic inflammation [19]; the lethality of the indomethacin-β-glucan combination was not confirmed by others [20], using several different types of β-glucans. The discussion of this experimental model, however, is outside the scope of this article and the reader is referred to original works.

β-glucans from *Lentinus edodes* inhibited lipopolysaccharide (LPS)-induced NO and tumor necrosis factor (TNF)α release by a macrophage cell line *in vitro* and gene expression of iNOS mRNA and TNFα mRNA [21]. An extract of the edible oyster mushroom reduced the release of TNFα and IL-6 by LPS-challenged monocyte *in vitro*, and also *in vivo* in mice challenged with LPS, after administration of the mushroom extract [22].

Two different β-glucan extracts from *Lentinus edodes* exerted different immunomodulatory activities: the commercial extract, containing higher amounts of α-glucans with respect to β-glucans, reduced the production of pro-inflammatory cytokines, transforming growth factor (TGF)-β and IL-10, and the oxidative stress-induced apoptosis, while the in-house extract attenuated late apoptosis [23].

An extract from *Agaricus blazei*, known to be pro-inflammatory *in vitro*, reduced cytokine levels in human blood of healthy volunteers after oral intake for several days of the mushroom extract [24].

The lentinan exerted a significant anti-viral activity on infected epithelioma cells, by both direct inactivation and inhibition of viral replication: the lentinan effect was attributed to the regulation of the innate immune responses and specific immunity, in addition to the down-regulation of the inflammatory TNF-α, IL-2, and IL-11 and up-modulation of IFN-1 and IFN-γ after the viral challenge [25]. The antiviral and anti-inflammatory effects of mushrooms have been recently reviewed and suggested as therapeutic potential to be developed against SARS-CoV-2 [26].

### 4.2. β-Glucans in Pre-Clinical Experimental Models

The reported [17,21,22,23,24], apparently discrepant, results on the immunomodulatory and anti-inflammatory effects of mushroom β-glucans may be partly explained by the great heterogeneity of mushrooms, different extraction techniques of β-glucans, and *in vitro* or *in vivo* models of testing, and confirm complex and not completely understood mechanisms.

Nevertheless, their potential clinical use is of great value and is being considered in different pre-clinical experimental models and in clinical studies. β-glucans from mushrooms effectively improved the immune response or survival in different experimental models of sepsis, such as pulmonary sepsis induced by intra-tracheal administration to rats of antibiotic-resistant *Klebsiella pneumoniae* [27], or cryptococcal infection in mice [28]. Lentinan exerted intestinal anti-inflammatory activity in a mouse model of colitis, through inhibition of IL-8 mRNA expression [29] and improved the intestinal barrier function in piglets, decreasing rotavirus-induced diarrhea [30].

### 4.3. β-Glucans in Infections and Allergy

The immunomodulatory effect of β-glucans (a syrup containing the pleuran β-glucan from *Pleurotus ostreatus* and vitamin C) has been tested versus vitamin C alone in a clinical trial in children with recurrent respiratory tract infections [28]: the respiratory symptoms were reduced, but the beneficial effect cannot be attributed to the β-glucans alone, rather should be related to a potentiating effect by β-glucan of vitamin C [31]. The Shiitake mushroom extract Lentinex^®^ given as a supplement in a cross-over, placebo-controlled trial in healthy elderly was safe and induced an increase in the number of circulating B-cells, without affecting any other immune parameter [32]. A randomized, double-blind, placebo-controlled study with pleuran, the β-glucan isolated from *Pleurotus ostreatus*, showed a significant reduction of peripheral blood eosinophilia and stabilized the levels of total immunoglobulin (Ig)E in serum in children with recurrent respiratory tract infections, suggesting a potential anti-allergic effect of mushroom-derived β-glucans [33].

Few studies on autoimmune diseases have been performed: AndoSan™, an extract derived from *Agaricus Blazei Murill* by more than 80%, was tested in inflammatory bowel disease, Crohn disease, and ulcerative colitis with modest results on inflammatory cytokines or clinical symptoms [34,35].

### 4.4. β-Glucans as Adjuvants in Oncological Disorders

**Figure 2:**
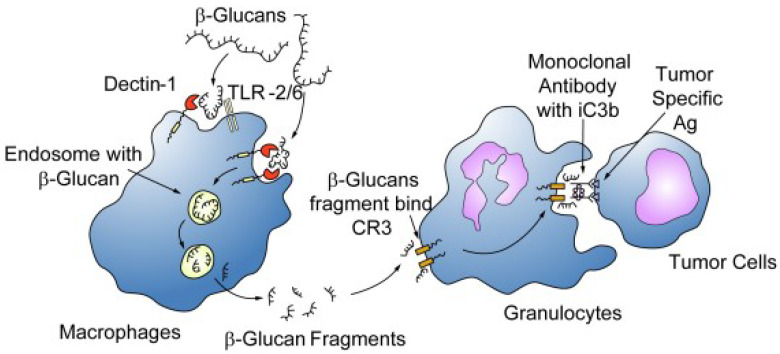
Schematic effect of β-glucans on immune and cancer cells. β-glucans are captured by the macrophages via the Dectin-1 receptor with or without TLR-2/6, internalized, fragmented into smaller fragments, carried to the marrow and endothelial reticular system and subsequently released. These small β-glucan fragments are eventually taken up by circulating granulocytes, monocytes or macrophages via the complement receptor (CR)3 and the immune responses turned include phagocytosis of the monoclonal antibody tagged tumor cells. From [37] with permission.

No direct cytotoxic effect on cancer cells was reported for β-glucans, but their adjuvant activity was shown in mice concomitantly treated with anti-tumor monoclonal antibodies, the dual treatment resulting in mammary and hepatic tumor regression significantly greater than each single treatment [36]. The adjuvant mechanism of immune system activation depends on the formation of an immune complex of β-glucans, after recognition as PAMP, with anti-β-glucan antibodies, which through immune effector cells and C3 phagocytes facilitates killing of tumor cells, as represented in Figure 2 [37].

The major roles of mushroom-derived β-glucans on cancer progression have been extensively reviewed [38]. A promising application of β-glucans is their use in cancer treatment, as adjuvant of conventional therapies. A number of clinical trials, phase 1 and 2 studies, registered on *clinicaltrials.gov* and listed in recent reviews [13,39,40] are ongoing on different tumor types, including pancreatic, head and neck cancer, non-small cell lung carcinoma, non-Hodgkin’s lymphoma, colorectal cancer, neuroblastoma, and triple negative breast cancer. Most clinical trials have been testing registered β-glucan extracts from yeast, such as Imucell^TM^ WBG or BTH1677 (Imprime PGG), in combination with PD-1 (programmed death receptor) blocking antibody or immune checkpoint inhibitors or with conventional treatments. Few of these studies have been completed and the results published so far support that these products are well tolerated and safe and could improve the remission rate as first line treatment, combined with conventional chemotherapy in non-small cell lung carcinoma patients [41,42], or with immunotherapy in colorectal cancer [43].

Within the mushroom β-glucans, lentinan has been tested in different clinical settings. A meta-analysis on 650 individual patient data showed that addition of lentinan to standard chemotherapy significantly prolonged the survival of patients with advanced gastric cancer in respect to chemotherapy alone [44]. Lentinan prolonged the survival of gastric cancer patients receiving fluoropyrimidine (S-1)-based chemotherapy; the ratio of granulocytes/lymphocytes was significantly higher in the patients receiving lentinan compared to the controls with chemotherapy alone, supporting an immunological effect of lentinan [45]. Zhang et al. [46] reviewed the randomized controlled trials published from 2004 to 2016, conducted in China to test lentinan in combination with chemotherapy in lung cancer patients. In the 38 trials analyzed with 3117 total participants, lentinan administered intravenously (1–1.5 mg/day for two to eight weeks) improved the patients’ quality of life and increased the overall response rate of chemotherapy (56% versus 43.3% of chemotherapy alone).

A recent systematic review of clinical trials on fungal β-glucans in cancer patients reports that on 16 trials with a total of 1650 patients the administration of β-glucans is safe and well-tolerated and that their administration concomitant with chemo or radiotherapy reduced the immune depression caused by such treatments and accelerated the recovery of white blood cell counts; however, these conclusions are still controversial, due to some not statistically significant findings and to a great diversity among trial methodologies or lack of information [47].

### 4.5. β-Glucans in Metabolic and Gastro-Intestinal Systems

The mushroom β-glucans are not digested in human gastrointestinal tract, as the enzymes secreted by intestinal brush border epithelial cells are unable to hydrolyze β-glycosidic bonds; within other properties, they speed up the transit of bowel contents, increasing fecal bulk and frequency, with possible beneficial properties of protection from irritable bowel syndrome, diverticular diseases, and colon cancer [13].

**Figure 3:**
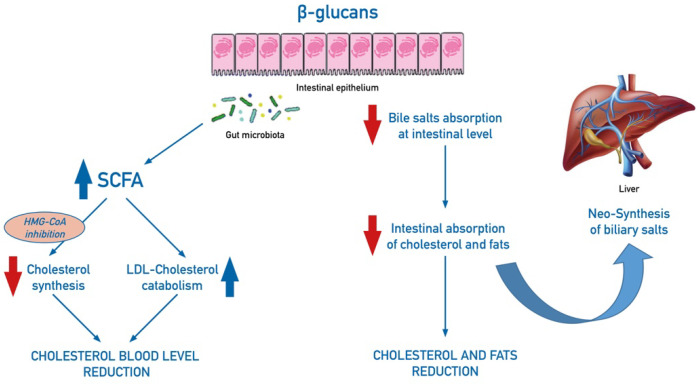
Schematic mechanisms of effect of dietary intake of β-glucans on cholesterol balance. β-glucans increase the production of short-chain fatty acids (SCFA) by intestinal microflora fermentation: SCFA inhibit cholesterol levels by acting on its synthesis, through inhibition of the hydroxymethylglutaryl-coenzyme A (HMG-CoA) reductase, and by increasing LDL-cholesterol catabolism. In addition, β-glucans, by forming a gel on the mucosal surface of the bowel, inhibit intestinal resorption of biliary salts, cholesterol, and fats and stimulate neo-synthesis of biliary salts in the liver.

β-glucans lower cholesterol levels, through different reported mechanisms, still not completely understood [48]. Acting as prebiotics, β-glucans has been associated to the production of short-chain fatty acids (SCFA) by intestinal microflora fermentation, which are able to inhibit cholesterol synthesis, by inhibition of the hydroxymethylglutaryl-coenzyme A (HMG-CoA) reductase, and to increase LDL-cholesterol catabolism. In addition, β-glucans, by forming a gel on the mucosal surface of the bowel, inhibit intestinal resorption of the bile salts and stimulate their neo-synthesis in the liver. As a consequence, increased biliary salts activate utilization of circulating cholesterol, thus reducing its level in the blood (Figure 3). Dietary fibers also bind lipids and cholesterol and decrease absorption at the intestinal level and increase the fecal excretion of these substances. Modulation of cholesterol-related genes by a glucan fraction of *Pleurotus ostreatus* was shown in a mice model [49].

#### 4.5.1. β-Glucans from Cereals and the EFSA Health Claims

The β-glucans from cereals, due to their 1,3 and 1,4 linkages, are recognized by the organism as non-digestible dietary fibers and exert different metabolic activities, which positively influence the cardiovascular system: they have been shown to lower cholesterol, triglycerides and apolipoprotein B in adult subjects; several trials have been reviewed in two meta-analyses, considering diets enriched with oat and barley β-glucans, respectively [50,51]; systolic and diastolic blood pressure were also reduced in mild or borderline hypertensive patients [52], and in hypertensive obese men and women [53], glycemic and insulinemic responses were reduced by oat β-glucans at increasing viscosity given with breakfast to healthy subjects, without modifying appetite or food intake [54].

A beverage enriched with 5 or 10 g of β-glucans from oats (but not from barley) administered for eight weeks decreased total cholesterol and postprandial glucose and insulin concentrations in hypercholesterolemic subjects [55]; several trials showed in type 2 diabetes patients reduced fasting blood glucose levels and glycosylated hemoglobin percentages upon supplementation with fibers or beta-glucans from cereals and an umbrella meta-analysis of meta-analyses and trials on type 2 diabetic receiving β-glucans or psyllium fibers summarizes these interesting results [56].

Within the different mechanisms at the basis of the reported cardiometabolic effects of β-glucans, changes of gut microbiota and stimulation of colon commensal bacteria, such as *Lactobacilli and Bifidobacteria* species, have been considered part of the final (beneficial) effects on bile acids, small-chain fatty acid signaling and cholesterol metabolism regulation and also on the immunomodulatory effects already reported in this review (see for review [57]).

In 2011 the European Food Safety Authority (EFSA) Panel on Dietetic Products, Nutrition and Allergies addressed “the scientific substantiation of health claims in relation to β-glucans from oats and barley and maintenance of normal blood LDL-cholesterol concentrations, increase in satiety leading to a reduction of postprandial glycemic responses, and digestive function” [58]. The LDL-cholesterol level lowering effect in the general population had been already accepted as a claim in 2009 by the same panel; to obtain a postprandial glycemic reduction, 4 g of β-glucans from oats or barley for each 30 g of available carbohydrate should be consumed by an individual who wishes that; the latter digestive function was not sufficiently defined [59].

#### 4.5.2. β-Glucans from Mushrooms

Similar beneficial physiologic effects on the cardio-metabolic system have been reported also for β-glucans from mushrooms in numerous *in vitro* cell systems and in animal experiments, with attention to the main mechanisms involved [60], while the studies in humans supporting these effects are scarce.

A recent systematic analysis reviewed the human intervention studies on *Pleurotus ostreatus* (or oyster mushroom), one of the most common edible mushrooms worldwide, eaten fresh or cooked or in dried form, on cardiometabolic parameters and reported some beneficial effects on glucose and lipid metabolism, and partly blood pressure [61]. The authors, however, considered with cautions the reported effects, due to the small number of subjects studied and to the trial designs not sufficiently controlled and adequate [61].

A β-glucan-enriched mixture, corresponding to 3.5 g/day of fungal β-glucans, obtained from Shiitake mushrooms and incorporated in three different commercial food creams, was given to hypercholesterolemic subjects, in a controlled, randomized, double-blind trial. After eight-week intervention, no changes in lipid- or cholesterol-related parameters were reported in either subjects receiving β-glucans or placebo; no differences were also observed in the concentrations of inflammatory cytokines (IL-1β, IL-6, and TNF-α) or oxidized LDL, at the end of the intervention in the two groups. On the other hand, interestingly, in the same trial the mushroom mixture modulated the microbiota differently from placebo, cholesterol and dietary fiber intake being the most associated factors to the observed microbiota variations [62].

Although suggestive and supportive of future larger studies, β-glucans from mushrooms, at variance to those from oats and barley, do not seem to beneficially affect lipid levels and glycemic responses in humans. Besides differences in trial designs, power calculations, and type of subjects included in the studies (healthy, hypercholesterolemic, diabetic subjects, and elderly), the different mushroom species used as source of β-glucans may have contributed to the uncertain findings. The chemical structure of β-glucans and the degree of branching with substituents or protein/peptide links define the molecule tridimensional conformation and may influence the water solubility and the biological activities of the β-glucans derived from different mushroom species.

The β-glucan content in different cultivars of *Lentinula edodes*, determined by a specific kit (Megazyme International, Ireland), ranged from 20 to 40% and from 33 to 58%, respectively, in the pileus and the stipe regions of the dried fruiting bodies [63]; these values of β-glucan content were confirmed by two subsequent studies detecting β-glucans in a large number of fruiting bodies of wild and commercial edible mushroom species, including the *Lentinula edodes* [64,65]. The latter study assayed β-glucans also with the Congo red method, which detects the β-glucans with a triple-helix chain conformation, possibly characterized by more efficient immunomodulatory activities [10]. Table 1 reports all glucan and β-glucan content in different species and parts of commercially cultivated mushrooms [64]. The α-glucan content in mushrooms was only a small percentage of total glucans, even less than 1.5% in some *Lentinula edodes* cultivars [63,64,65].

## 5. Mushrooms beyond β-Glucans

Considering mushrooms as a food, few epidemiological data are available.

Human studies on the association between edible mushroom consumption and cardiovascular risk were recently collected in a systematic review [66], which included analyses from seven observational and prospective cohort studies and randomized clinical trials, heterogeneous for interventions, outcomes, and timing. The results demonstrated no significantly different risk of total cardiovascular disease (defined as fatal cardiovascular disease and non-fatal myocardial infarction) in individuals consuming more than five servings of mushrooms per week compared to those who consumed less than one portion per month, with a follow-up greater than 2 million person-years, a finding resulting from a single large prospective study [67]; in type 2 diabetes, a small reduction of glycated hemoglobin was reported, but only based on observational studies and not confirmed by intervention trials; blood pressure reduction and favorable changes of lipid profiles were also suggested by weak evidence [66].

A randomized parallel controlled trial on hyperlipidemic subjects compared the effect of two different Japanese diets recommended for six months through periodic face-to-face nutrition education and counselling: the diet which recommended to consume more healthy foods, including mushrooms, resulted in a greater decrease of serum LDL-cholesterol, triglyceride, and insulin in respect to the partial Japanese diet [68].

A large cross-sectional study on 24,236 Chinese adults, significantly associated a higher mushroom intake with lower prevalence of non-alcoholic fatty liver disorder (NAFLD), an original observation on a condition of high cardiovascular risk, which however needs to be prospectively confirmed [69].

The dietary intake of mushrooms and selenium, mainly contained in mushrooms, were studied in subjects without a history of diabetes or cardiovascular disease from the large Italian Moli-sani cohort in relation to blood glucose levels: both mushrooms and selenium resulted to be independently associated with glucose levels and high intake with higher prevalence of diabetes [70]; the authors suggested that mushrooms may contain other elements, besides selenium, responsible for the negative effect on glycemia.

On the other hand, the National Health and Nutrition Examination Survey (NHANES) 2011–2016 reported that addition of a serving of mushrooms to the diet would increase several micronutrients (dietary fibers, copper, phosphorus, potassium, selenium, zinc, riboflavin, niacin, choline, iron, thiamine, folate, and vitamin B6) in both adolescents and adults, but had no impact on energy, carbohydrate, fat, or sodium; as a consequence, the intake of a serving (84 g) of commonly consumed mushrooms could partially correct an inadequate intake of the reported micronutrients, which is associated with adverse health effects. In the same survey, the addition of UV light-exposed mushrooms decreased the population inadequacy for vitamin D [71]. It is worth recalling that mushrooms are also a rich source of ergosterol, a precursor of vitamin D, and an important source of vitamin D itself, when mushrooms have been exposed to UV light [72]. In a previous analysis of NHANES 2001–2010, it was reported that mushrooms are rich sources of several micronutrients and that adults consuming mushrooms had higher diet quality than those who did not [73].

## 6. Conclusions

The polysaccharides β-glucans are important constituents of fungi cell walls; in the last years, their health-related effects have been studied in cellular and experimental models, to add scientifically-based knowledge to the long lasting anecdotal beneficial effects and to clarify their mechanisms. 

The immunomodulatory and anti-inflammatory effects of β-glucans from different mushrooms’ species have been reported and the molecular mechanisms of immune recognition by different white blood cells, have been described as mediated by specific receptors and cell signaling pathways, resulting in inflammatory immune gene expression and production of inflammatory mediators. On the other hand, in particular conditions, β-glucan extracts have been shown to modulate or reduce an ongoing inflammatory response stimulated by inflammatory stimuli, such as bacterial LPS.

These recognized activities of β-glucans from mushrooms, but also from other sources, have been considered for potential clinical use in different pathological conditions, such as infections of respiratory tract recurrent in children, or complications of major abdominal or thoracic surgery. Another promising application of β-glucan extracts is cancer treatment, as adjuvant of conventional therapies. 

Mushrooms may affect the cardiovascular system, through their β-glucan content, but also via a number of components, such as bioactive phenolic compounds, vitamins, and mineral elements, of which mushrooms are also rich.

Although the mechanisms and heterogenous effects of the different extracted materials are not completely understood, the immunomodulatory and anti-inflammatory activities, as well as the cardiovascular protective effects are being tested in clinical studies, mainly using β-glucans from mushrooms, or concentrated extracts as nutraceuticals or in view of potential drug discovery.

These scientific and mechanistic findings more consistently support the beneficial activities of mushrooms and indications about the fungi species or molecular structures most active on the different health aspects.

The future development of mushroom knowledge would hopefully substantiate the role of this food ingredient in the context of a healthy diet, in view of preserving human health and preventing disease through a correct lifestyle.

## Figures and Tables

**Table 1:** All glucans and β-glucan content in different commercially cultivated mushrooms.

Mushroom *Genus Species* (Common Name) Part of the Fruiting Body	All Glucans (g/100 g Dry Matter)	β-Glucans (g/100 g Dry Matter)
*Agaricus bisporus* (white mushroom) cap	10.1 ± 2.2	8.6 ± 2.4
*Agaricus bisporus* (white mushroom) stalk	15.0 ± 5.0	12.3 ± 4.1
*Agaricus bisporus* (brown mushroom) cap	12.3 ± 4.5	8.8 ± 3.0
*Agaricus bisporus* (brown mushroom) stalk	14.6 ± 4.9	10.1 ± 2.2
*Lentinula edodes* (shiitake) cap	20.5 ± 6.0	20.0 ± 6.2
*Lentinula edodes* (shiitake) stalk	26.7 ± 4.0	25.3 ± 4.4
*Cantharella cibarius* cap	25.3 ± 1.7	24.0 ± 1.7
*Cantharella cibarius* stalk	28.5 ± 2.3	27.0 ± 2.5
*Pleurotus ostreatus* (oyster mushroom)	25.6 ± 1.6	24.2 ± 1.6
*Pleurotus eryngii* (king oyster mushroom)	19.2 ± 1.6	15.3 ± 1.7
*Pleurotus citrinopileatus* (golden oyster mushroom)	18.3 ± 1.5	15.5 ± 1.4
*Pleurotus pulmonarius* (lung oyster mushroom)	19.4 ± 0.6	17.5 ± 0.6
*Pleurotus djamor* (pink oyster mushroom)	23.6 ± 1.1	21.7 ± 0.8

Data are reported as means and SD; all glucans are the sum of α- and β-glucans. Data from [64].

## Data Availability

Not applicable.
